# Real-World Data as Real Evidence Showing Real-World Outcomes

**DOI:** 10.15586/jkc.v12i2.404

**Published:** 2025-05-14

**Authors:** Ulka Vaishampayan

**Affiliations:** Department of Medicine, University of Michigan, Michigan, United States

## Introduction

Metastatic/advanced kidney cancer currently has at least four US Food and Drug Administration (FDA) approved regimens that have established overall survival benefit as frontline therapy. Ipilimumab and nivolumab (ipi + nivo) is the only regimen consisting of a combination of immune checkpoint therapies; the other regimens consist of a combination of vascular endothelial growth factor-tyrosine kinase inhibitor (VEGF-TKI) therapy and immune checkpoint inhibitor (ICI). Each of these new regimens have been compared to the standard therapy at that time—a single-agent VEGF-TKI (sunitinib). As the two strategies of immune checkpoint therapy alone versus combination with VEGF-TKI have not been compared to each other, clinicians cannot make an evidence-based choice between the four regimens. On the National Comprehensive Cancer Network (NCCN), t all regimens have category 1 preferred status as all have independently shown improved survival and efficacy in comparison to sunitinib. It is unlikely that randomized trials with noninferiority design will be conducted comparing the two strategies.

Currently, a French trial is in progress evaluating this comparison; however, it is entirely possible that novel regimens will have replaced the standard of care by the time the results are available. In situations like this, the real-world evidence (RWE) can be an important complementary piece of verification enabling the clinician to make decisions on their real-world patients.

RWE analysis is a retrospective study that can be rightfully critiqued for the inherent biases of this methodology; it should also be interpreted with care. The FDA provides guidance about the utility, application, and conduct of RWE generation and analysis (1). The FDA framework document states that RWE can be utilized to improve the efficiency of clinical trials. There are some valuable assets to RWE reporting such as large sample size, inclusion of diverse populations, as well as patients with decreased performance status or multiple associated comorbidities. In summary, RWE provides information on clinical outcomes with contemporary therapies in the patient population that typically would not have qualified as candidates for a prospective randomized clinical trial.

The current study by Ostrowski et al. (2) utilized the FlatIron database that collected patient information from academic and community practices nationwide from electronic health records. The study included metastatic clear cell renal cancer patients treated with immune checkpoint-based combination therapy between 2016 and 2023. Primary outcomes of the study were real-world time to next therapy (rwTTNT) and overall survival (rwOS). The comparisons between ipi + nivo regimen and VEGF-TKI + immune checkpoint therapy were conducted in the context of propensity score (PS) matching weighted analysis. The results showed no difference in overall survival outcomes, but rwTTNT was significantly shorter with Ipi + Nivo than with ICI + TKI (HR 0.78, 95% CI 0.68–0.89; p < 0.001). After the propensity score matching weighted analysis, the median rwTTNT and median OS, respectively, were 7.8 months and 28.8 months for patients treated with ipi + nivo compared to 13.1 months and 25 months for patients treated with ICI + TKI. It is sobering to note that although the median progression-free survival (PFS) is comparable, the median overall survival (OS) is much shorter in the real-world study than the median survival of 52.7 months, noted in the CheckMate 214 prospective randomized trial of ipi + nivo and about 48 months noted in the ICI + TKI trials. The median OS here with ICI-based regimen is similar to that seen with sunitinib (control arm) in the prospective randomized trials. This study increases awareness that there is likely to be a large gap between the real world and the clinical trials data and outcomes, and highlights the real importance of RWE.
